# Robot-Assisted Perineal Radical Prostatectomy in a Post-Kidney Transplant Recipient

**DOI:** 10.1089/cren.2017.0119

**Published:** 2018-02-01

**Authors:** Volkan Tugcu, Abdulmuttalip Simsek, Ismail Yigitbasi, Mustafa Gurkan Yenice, Selcuk Sahin, Ali Ihsan Tasci

**Affiliations:** Department of Urology, University of Health Sciences, Istanbul Bakirkoy Dr. Sadi Konuk Research and Training Hospital, Istanbul, Turkey.

**Keywords:** robotics, perineal, transplantation, prostate cancer, radical, prostatectomy

## Abstract

***Background:*** After almost two decades, transabdominal robotic radical prostatectomy techniques have been fully developed and are widely practiced by many robotic urologists. Recently, a transperineal robotic radical prostatectomy, a technique not yet popular to many, was introduced as an alternative approach in patients with previous abdominal surgery. Here, we present our unique experience with robotic perineal radical prostatectomy (r-PRP) on a kidney transplant recipient.

***Case Presentation:*** A 71-year-old man who had a kidney transplant 4 months previously was diagnosed with prostate cancer (PCa) and underwent r-PRP using the da Vinci Xi robotic system. The operative time was 110 minutes and blood loss was minimal. After the perineal drain was removed on postoperative day 3, the patient was discharged. The urethral catheter was subsequently removed on postoperative day 8. Pathologic analysis revealed localized PCa with negative surgical margins.

***Conclusion:*** The r-PRP offers all the advantages of minimally invasive surgery. Moreover, in a kidney transplant recipient, it provides additional benefits, such as avoidance of allograft vascular and ureteral injuries, while maintaining an equivalent oncologic efficacy and surgical safety compared with its transabdominal counterpart.

## Introduction

Aradical prostatectomy is the gold standard for the treatment of localized prostate cancer (PCa). There have been many advances in the field of radical prostatectomy and several techniques have been described.

Kaouk et al.^1^ described the robotic perineal radical prostatectomy (r-PRP) at the Cleveland Clinic in 2016. They performed this technique first in a cadaver model, we then performed this technique on 15 patients and presented our results.

This new r-PRP technique has many advantages, one of which is that it is performed in a different compartment than the abdominal cavity, and the probability of causing anatomical or physiologic damage to the intestines is low. In the present case, we performed r-PRP on a patient who had previously undergone a renal transplant. In robot-assisted laparoscopic radical prostatectomy (RALRP) and other laparoscopic methods, the abdomen is insufflated with carbon dioxide. Depending on the gas pressure, collapse of the renal vein, a kidney vascular circulation disorder, and acute tubular necrosis may develop. When the bladder is dissected with transperitoneal RALRP or laparoscopic methods, the transplanted kidney in the extraperitoneal compartment may be damaged. In the open or laparoscopic RALRP, during the dorsal venous and endopelvic fascial dissections, the transplanted ureter and ureterovesical anastomosis may be damaged, thus causing serious problems.

## Presentation of Case

**FIG. 1.**
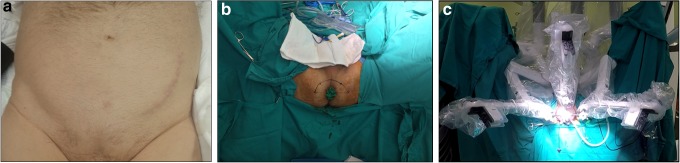
**(a)** Transplant (left) incision scar. **(b)** Placing a sterile glove on the rectum, semilunar incision lines. **(c)** Docking of the robotic system.

A 71-year-old man developed end-stage renal failure because of polycystic kidney disease. The patient received hemodialysis treatment for 13 years. He also had a history of a laparoscopic cholecystectomy. A renal transplant from a cadaveric donor was performed on the patient with no additional diseases at another center in May 2017 (Fig. 1a). The patient's urethral catheter was removed during the postoperative period and the patient was unable to urinate, thus acute urinary retention occurred. Laboratory tests showed that the prostate-specific antigen level was 3.77 ng/cc. On multiparametric MRI, a PIRADS IV lesion (5 mm) was detected on the right lateral side of the peripheral zone of the prostate at the midgland level. A transrectal ultrasound-guided prostate biopsy was performed on the patient, who had a prostate volume of 55 cc. The pathology result was a 4 + 3 on a 2/10 focus according to the Gleason score with no extraprostatic spread. No lymphadenopathy was detected in the region within the scanned area. Based on the Partin nomogram, there was no need for a lymph node dissection. The patient underwent r-PRP using the da Vinci Xi 3 arm robotic system.

The patient was placed in an exaggerated lithotomy and 15 degrees Trendelenburg position. At the start of the operation, a sterile glove was placed on the rectum and the tips were fixed to the perianal region using a silk suture. The perioperative digital rectal examination was performed with a sterile glove to avoid damaging the rectum (Fig. 1b). A 6-cm semilunar incision was made between the two tuberculum ischiadica. The posterior fibers of the perineal body were dissected. The bilateral ischioanal fossa was bluntly dissected and a gap was formed. The dissection proceeded until the membranous urethra. The dissection ceased after the apex of the prostate appeared. GelPOINT^®^ (Applied Medical, Rancho Santa Margarita, CA) was inserted into this cavity. Trocars were placed in the GelPOINT for the camera, an 8-mm trocar was placed at the 12 o'clock position. The other two trocars were placed at the 5 and 7 o'clock positions. For assistance, a 10-mm trocar was placed at the 6 o'clock position (Fig. 1c).

**FIG. 2.**
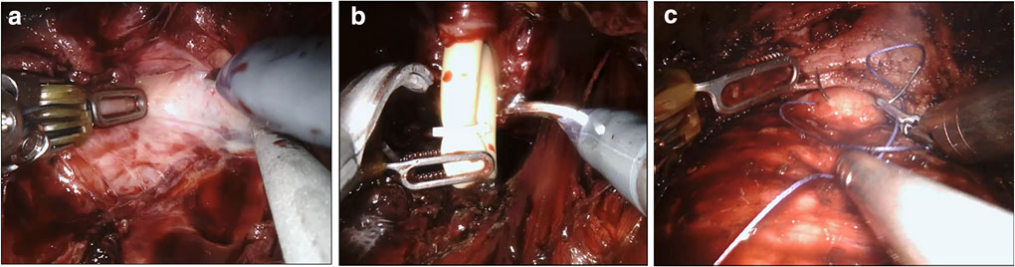
**(a)** Lateral lobe dissection. **(b)** Placing the clip in and cutting the urethral catheter. **(c)** Strap suture in the middle lobe.

**FIG. 3.**
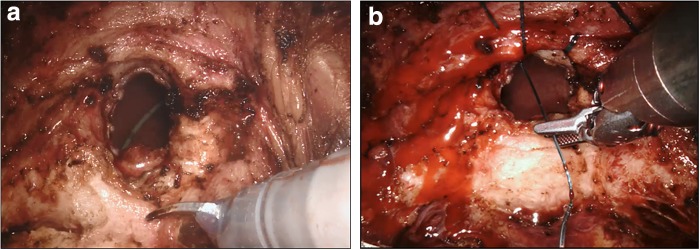
**(a)** Double-J catheter of the transplanted kidney. **(b)** Vesicourethral anastomosis.

After insufflation started, dissection was initiated from the apex of the prostate and extended to the lateral lobes (Fig. 2a). The dissection was then extended to the deep plane. Denonvilliers' fascia was then identified and the seminal vesicle compartment was reached. The seminal vesicles were fully and bilaterally dissected and released. The membranous urethra was cut, and the urethral catheter was removed from the area of the incision. A Hem-o-Lock^®^ clip (Teleflex Medical, Research Triangle Park, NC) was placed in the urethral catheter and, with laparoscopic scissors, a urethral catheter was cut from the upper part of the clip (Fig. 2b). The urethral catheter was then used as a traction device. The prostate pedicles were dissected and cut with a Harmonic Robotics^®^ ultrasonic device. The prostate pedicles were released, and the dissection proceeded through the membranous urethra toward the base of the prostate. The dissection was extended toward the bladder neck with monopolar scissors while sparing the dorsal vein complex. The bladder neck was opened, and the middle lobe of the prostate was monitored. To cut the bladder neck from behind the middle lobe, a strap suture was placed in the middle lobe of the prostate to function as a traction device (Fig. 2c). The prostate was fully dissected from the bladder and the neoureteral orifice and Double-J catheter of the transplanted kidney were then observed in the bladder (Fig. 3a). The robot was undocked to remove prostatic tissue from the surgical region and then redocked. A vesicourethral anastomosis was started and two 3/0 V-Loc™ (Covidien, Mansfield, MA) sutures were used (Fig. 3b). An anastomosis was performed with two sutures, one of which continuously proceeded clockwise to the 6 o'clock position starting from the 12 o'clock position, and the other continuously proceeded counterclockwise to the 6 o'clock position starting from the 12 o'clock position. A Jackson–Pratt drain was placed in the operated area. The surgical duration was 110 minutes, the volume of blood loss was 60 mL, and no intraoperative complications were detected. Peroperative and postoperative urine output was within normal limits. Postoperative creatinine level was 1.2 mg/dL. There were no postoperative complications. The drain catheter was removed on postoperative day 3 and the patient was discharged. The urethral catheter was removed on postoperative day 8. The Gleason score grade of the pathology report was 3 + 4. No tumor was found within the surgical margins.

## Discussion and Literature Review

The PCa incidence has increased in renal transplant patients compared with the normal population. In these patients, who also receive immunosuppressive treatment, the incidence of various malignant diseases has also increased. The rate of PCa in patients who have had a renal transplant was reported to be 0.63%–0.75%.^2^ No consensus has been reached as to whether these patients, who had both a renal transplant and developed PCa, should be actively followed up. Although radiotherapy is an option for localized PCa, the presence of the transplanted kidney at the irradiation site and the higher probability of developing adverse effects compared with the normal population require a thorough evaluation before opting for this method. Although there are no reports of graft loss after radiotherapy, the adverse effects after radiotherapy should be effectively managed.^3^ In these renal transplant patients who developed localized PCa, a radical prostatectomy is the gold standard.

When performing a radical prostatectomy with robotic, laparoscopic transperitoneal, or extraperitoneal methods, carbon dioxide is insufflated into the intra-abdominal cavity. Therefore, particularly because of the gas pressure, renal blood flow may be reduced and the glomerular filtration rate may decrease. In the r-PRP method, insufflation is performed in another compartment; thus, kidney functions remain unaffected. In our case, peroperative and postoperative urine output and creatinine levels were within normal limits.

The risk of damaging the transplanted kidney is high in RALRP or laparoscopic transperitoneal radical prostatectomy when cutting the peritoneum and dissecting the bladder. Despite careful dissection, it is difficult to protect the kidney anatomy and renal parenchyma, and vascular structures could be seriously damaged.

Carvalho et al.^4^ found that of 2742 patients who had undergone a renal transplant between 1980 and 2016, 20 had PCa. An open retropubic radical prostatectomy was performed on 17 of these patients, and 10 patients had a bilateral pelvic lymph node dissection in addition to the radical prostatectomy. No perioperative complications were reported. During the postoperative period, the graft loss rate was 35%. One patient had a transplantectomy.^4^ In our case, perioperative and postoperative graft functions were normal. Graft functions of patients were followed regularly.

Another important issue is the protection of the ureter of the transplanted kidney. During the dorsal vein dissection and endopelvic fascia dissection in open, laparoscopic, and RALRP, the ureter of the transplanted kidney can be damaged. At the same time, after the completion of a radical prostatectomy, a urethrovesical anastomosis must be carefully performed.

With r-PRP, we performed the operation below the bladder neck level and without opening the endopelvic fascia. This method does not involve the ureter of the transplanted kidney in any way. Thus, a radical prostatectomy and anastomosis can be performed safely. Moreover, with r-PRP, there is an earlier mobilization and an earlier return to daily life. An early recovery prevents the functions of the transplanted kidney from being affected.

## Conclusion

The r-PRP can be safely performed at experienced centers. With the advantages provided by this method, a radical prostatectomy can be safely performed while maintaining the graft function in patients with PCa who have previously undergone a renal transplant.

## Abbreviations Used

PCa: prostate cancer
RALRP: robot-assisted laparoscopic radical prostatectomy
r-PRP: robotic perineal radical prostatectomy
